# Oral lichen planus and lichenoid lesions - challenges and pitfalls for the general dental practitioner

**DOI:** 10.1038/s41415-024-7063-y

**Published:** 2024-02-23

**Authors:** Rebecca Binnie, Marianne Louise Dobson, Anna Chrystal, Karolin Hijazi

**Affiliations:** 4141528367001 Institute of Dentistry, School of Medicine, Medical Sciences and Nutrition, University of Aberdeen, Aberdeen, AB25 2ZR, UK; 4141528367002 Oral Medicine Department, Dundee Dental Hospital and Research School, Dundee, DD1 4HR, UK

## Abstract

Lichen planus is a chronic, mucocutaneous inflammatory condition which, due to its prevalence, will be familiar to the dental profession. However, diverse forms of presentation, important differential diagnosis, potential malignant change and monitoring requirements often result in challenges for those in primary care. This paper looks to examine these challenges and provide information to support those who are involved in recognition and management of patients with lichen planus.

## Key points

- Refer any suspected oral lichen planus or lichenoid lesion to specialist services unless competent to manage in practice and if appropriate.
- Treat symptomatic patients with first line topical corticosteroid preparations for short-term management and as directed by specialist services for long-term management. For lichenoid contact reactions, engage with specialist requests to replace amalgam restorations.
- General dental practitioners should have awareness of the risk of malignant transformation associated with oral lichen planus, monitor patients and alert specialist services to any clinically significant changes.

## Introduction

Lichen planus (LP) is a mucocutaneous chronic inflammatory condition which can affect the oral cavity, skin and genitalia, as well as other extra-oral sites. LP affects just under 1% of the general population.^1^^,^^2^ Although it can occur at any age, it predominantly affects adults over 40 years old.^2^

While oral lichen planus (OLP) is a chronic inflammatory disorder of unknown aetiology, oral lichenoid lesions (OLLs) develop in response to an extrinsic agent.^3^

Lichenoid inflammation is characteristic of both OLP and OLLs: a band-like infiltration of lymphocytes at the epithelial-connective tissue interface with associated epithelial basal cell damage and saw-tooth-shaped epithelial rete ridges.^4^ While some clinical and subtle histological differences exist, differentiating between OLP and OLLs can be challenging, which has led to debate (even among specialists) in terminology.^5^ OLP and OLLs present routinely to oral medicine (OM) departments, but their prevalence means they will also present to primary care, albeit uncommonly. It is important that general dental practitioners (GDPs) have an appropriate level of knowledge regarding the diagnosis and management of OLP and OLLs as patients with these conditions can present with significant challenges.

Professional monitoring is required for OLP and OLLs in view of their status of oral potentially malignant disorders.^6^ Varied presentations, important differential diagnoses and difficult management often require referral to secondary care.

## Aetiology

### Oral lichen planus

LP is a disorder of unknown aetiology affecting mucous and serous membranes. *In vitro* and *ex vivo* experimental studies suggest that both CD4+ and CD8+ T-cell responses play a role in the pathogenesis.^7^^,^^8^ Aetiology and disease course are likely multifactorial involving genetic and environmental factors.^7^

LP has been associated with other autoimmune systemic disorders, such as thyroid disease, ulcerative colitis and myasthenia gravis.^9^^,^^10^ Lichenoid inflammation has been reported in paraneoplastic mucosal disease and Good's syndrome.^11^^,^^12^ Associations have been reported with certain liver diseases and hepatitis B vaccination.^13^ Conflicting findings have been reported with regards to a relationship between LP and hepatitis C but with an overall larger body of literature supporting a positive association between LP and hepatitis C, including a cohort study in 1,557 patients.^14^^,^^15^ On this basis, hepatitis C testing for LP patients has been recommended at a minimum in high-prevalence countries and high-risk patients.^14^

### Oral lichenoid lesions

OLL is an umbrella term used to describe a diverse range of conditions where the clinical or histological presentation is compatible with OLP but not typical.^16^ Notwithstanding the lack of universally agreed clinicopathological criteria, OLLs broadly include:

1. Lesions caused by a reaction to exogenous substances:
  Oral lichenoid contact reactions caused by dental materials (in particular amalgam)
  Lichenoid drug reactions (Table 1)

**Table 1:** Examples of common systemic drugs associated with lichenoid drug reactions (non-exhaustive list)

**Drug class associated with lichenoid drug reactions**	**Examples**
ACE inhibitors^19^	Enalapril, captopril, ramipril,* lisinopril*
Beta-blockers^19^	Atenolol*, oxprenolol, propranolol,* bisoprolol*
Disease-modifying antirheumatic drugs^20^	Penicillamine
	Sulphasalazine
	Gold salts
	Hydroxychloroquine
Diuretics^20^	Hydrochlorothiazide
	Furosemide
	Spironolactone
Non-steroidal anti-inflammatories^19^	Naproxen*
	Ibuprofen*
	Diclofenac
	Indomethacin
Antifungals^20^	Ketoconazole
Hypoglycemics^20^	Metformin*
Anticonvulsants^19^	Carbamazepine
Biologic agents^19^	Tumour necrosis factor-alpha antibodies (infliximab, adalimumab)
	PD1/PD-L1 antibodies (pembrolizumab)
	Interferon-alpha
Tyrosine kinase inhibitor^20^	Imatinib
Others	Allopurinol, methyldopa^19^
Key: * = Drugs commonly seen in primary care
  Oral lichenoid contact reactions caused by cinnamon according to a relatively small body of evidence^4^^,^^17^^,^^18^
2. Conditions mimicking LP such as oral graft versus host disease.

## Challenges in presentation

### Extra-oral manifestations

**Fig. 1:**
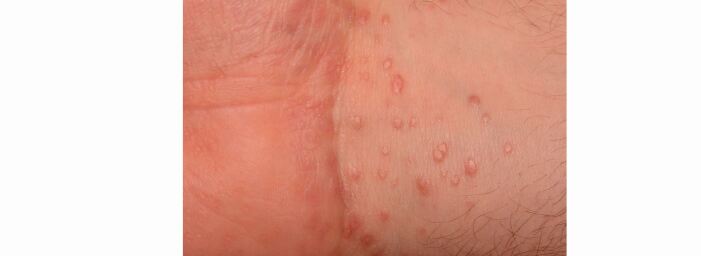
Cutaneous lichen planus affecting the wrist flexor surface

Cutaneous lesions are the most common extra-oral presentation of LP and affect around 50% of patients who present initially with oral symptoms. However, patients presenting initially with cutaneous LP are less likely to have oral lesions.^21^ The most common sites for cutaneous LP are the flexor surface of the wrists (Fig. 1), lower legs/ankles, trunk and lower back.^22^ Cutaneous LP presents as a pruritic rash of shiny, flat-topped papules with a purple hue and superimposed white lacy lines (‘Wickham striae').

Other extra-oral sites include the genitals, nails, scalp and less commonly, the larynx, oesophagus, cornea and conjunctiva.^23^^,^^24^ Genital lichen planus is seen in up to 57% of women with OLP.^25^ Vulvovaginal-gingival syndrome is a severe form of lichen planus, often more resistant to first-line treatment, that can result in scarring and strictures (Box 1).^26^

Box 1 Extra-oral manifestations

- GDPs should question patients presenting with suspected OLP about signs and symptoms of possible extra-oral manifestations
- GDPs should refer patients with possible multi-site involvement to their local secondary care unit (eg OM). In the meantime, patients should be advised to see their general medical practitioner
- Patients with multi-site involvement will require multidisciplinary management
- Other immune-mediated mucocutaneous conditions should be considered in the differential diagnosis of multi-site presentations. The diagnosis should be confirmed in a specialist setting.

### Diversity of clinical presentation of oral lichen planus

The diversity of clinical presentations of OLP can make clinical recognition challenging.

There are six presentations, which describe different clinical appearances and disease phases.^27^ A patient can have multiple presentations at any one time:

- Reticular - a common presentation of white line lacy patterns caused by epithelial hyperkeratosis. Small papules can also be present
- Atrophic - areas of erythematous mucosa caused by epithelial thinning
- Ulcerated/erosive - partial or full thickness loss of stratified epithelium presenting as erythematous areas, with or without a yellow surface and non-erosive reticular periphery
- Plaque-like - homogenous and well-demarcated white patches often affecting the buccal mucosa and dorsum of tongue
- Bullous - subepithelial blisters, often affecting the gingivae, that rupture to reveal areas of ulceration
- Desquamative gingivitis - a clinical descriptor of inflamed, erythematous, smooth gingivae. OLP is the most common cause of desquamative gingivitis.

**Fig. 2:**
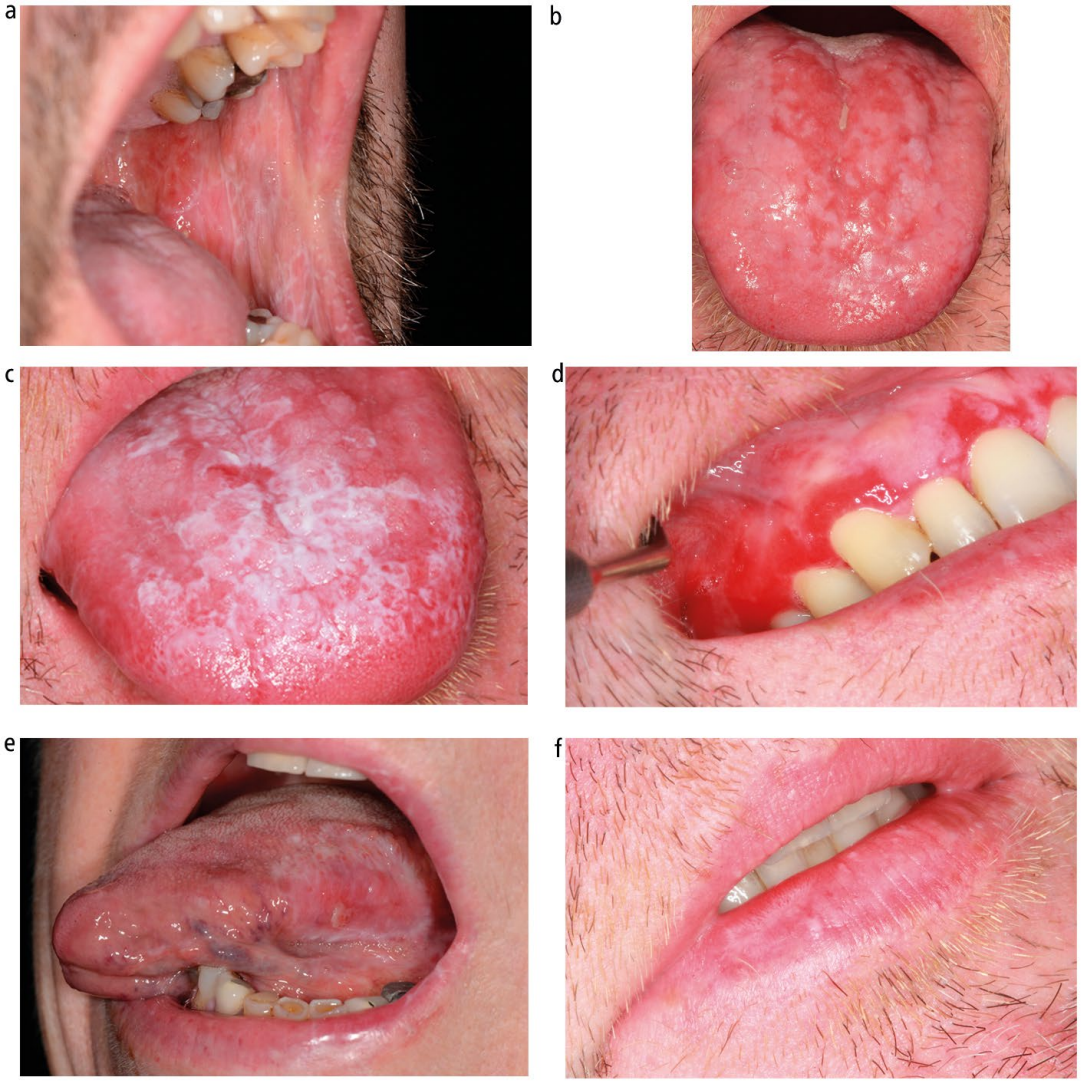
Clinical presentations of OLP. a) Reticular. b) Erosive/atrophic. c) Plaque-like. d) Desquamative gingivitis. e) Amalgam-related lichenoid reaction. f) Lichen planus of the lip

Different clinical presentations in comparison with LP of the lip and amalgam-related OLLs are shown in Figure 2.

The most common presentation of OLP is bilateral, symmetrical, white, reticular striations affecting the buccal mucosa. The tongue and gingivae are the next most common sites.^28^ Careful consideration is required in cases of desquamative gingivitis and the bullous presentation as these are also features of other immune-mediated mucosal diseases. The palate is rarely involved in OLP and LP-like lesions affecting this site should prompt consideration of an alternative diagnosis for example, lupus. (See ‘other conditions mimicking oral lichen planus' section).^29^^,^^30^

### Presentation of oral lichenoid lesions

OLLs can present within the same clinical spectrum as OLP, making differentiation between the two conditions challenging. In the case of lichenoid contact hypersensitivity associated with a dental material such as amalgam, the OLLs are in direct contact with the causative restoration.^31^ The lesion(s) often resolve or improve on removal of the causative factor, for example, replacement of the amalgam restoration with an alternative material.^32^

With regards to OLLs induced by medications, a recent review found the mean age of patients with lichenoid drug reactions to be 58.5 years.^33^ The most frequently reported causative drug were checkpoint inhibitors (anti-PD1/PD-L1 antibodies) and the median time from discontinuing the drug to resolution was 15.7 weeks. Naturally, withdrawal of the culprit drug often requires careful consideration of the risk and benefits and may not always be a practical option, for example, in the context of cancer treatment (Box 2).^33^

Box 2 Oral lichenoid lesions

- OLP shares similar clinical and histopathological features with OLLs with no current universal features to distinguish between them
- OLP has an unknown aetiology and no cure
- OLLs are due to a causative agent which often resolves following removal of offending agent, for example, drug exposure or local contact hypersensitivity reaction
- Thorough history and examination are essential to support diagnosis.

### Other conditions mimicking oral lichen planus

**Fig. 3:**
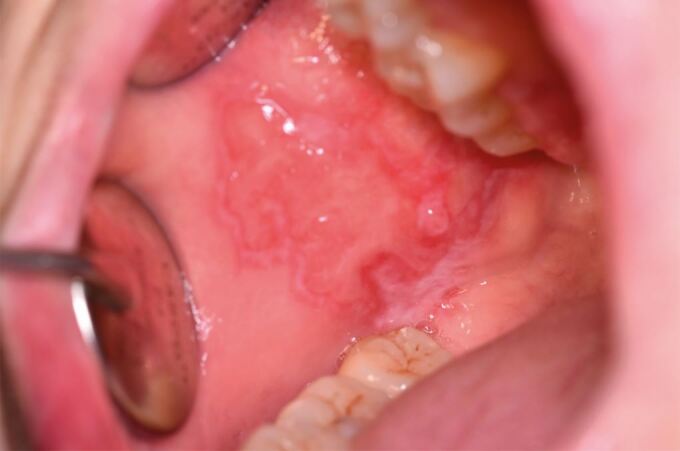
Oral lupus erythematosus of the buccal mucosa

**Fig. 4:**
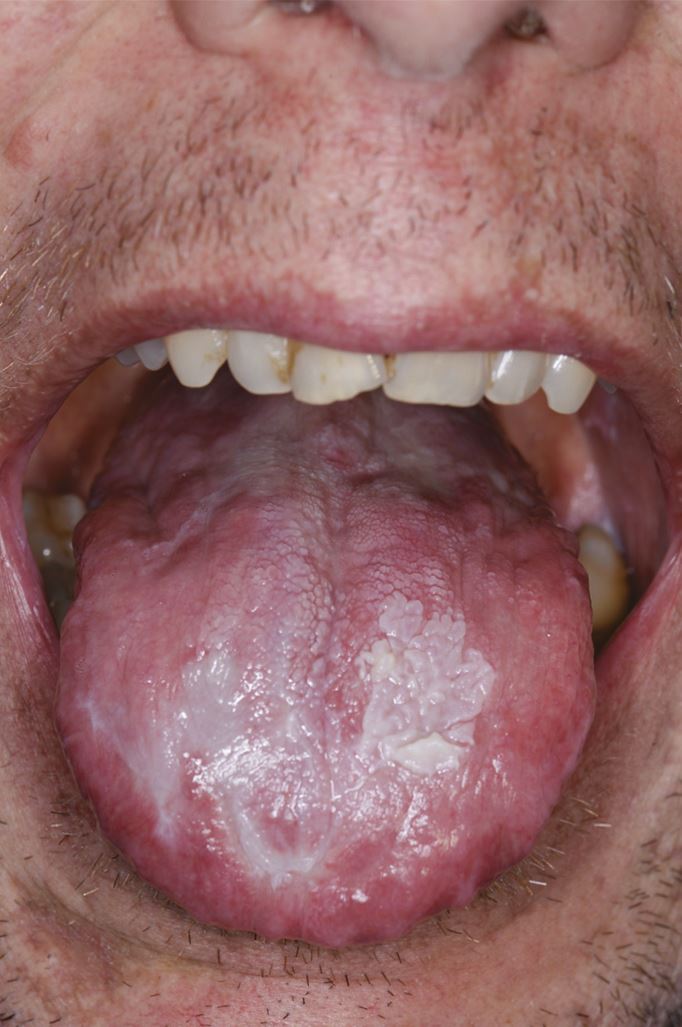
Chronic oral graft versus host disease lesions on the tongue

Oral lupus erythematosus can present similarly to OLP, with atrophic or erosive patches surrounded by white striations in a ‘sun-ray' appearance. The buccal mucosa, palate and lips are most commonly affected in oral lupus (Fig. 3).^16^ Oral graft versus host disease presents in patients receiving allogenic stem cell transplants. The clinical appearance and histological characteristics of chronic oral graft versus host disease are indistinguishable from OLP^27^ (Fig. 4). Both oral lupus erythematosus and graft versus host disease are oral potentially malignant disorders and require surveillance.^16^

LP-like lesions can be seen in paraneoplastic mucosal disease as described in the S2k guidelines on the management of paraneoplastic autoimmune multiorgan syndrome.^34^ Suspicion of multi-system disorders should be raised in cases of atypical clinical presentations, and/or constitutional symptoms.

### Disease severity and symptoms

While some OLP/OLL cases are asymptomatic, other patients report significant symptoms associated with an adverse impact on quality of life.^35^ Patients may describe a variety of symptoms (Box 3). Patient-reported symptoms and the impact on daily activities does not necessarily correlate with clinical disease severity. Patient-centered tools for measuring the impact of chronic oral mucosal disease on quality of life have been developed, for example, the Chronic Oral Mucosal Diseases Questionnaires (COMDQ).^36^

Box 3 Overview of potential symptoms of OLP/OLLs

- Spontaneous discomfort or pain
- Discomfort or pain on consumption of strongly flavoured food/drinks
- Feeling of swelling
- Discomfort/pain on toothbrushing
- Discomfort/pain speaking
- Dysgeusia
- Areas of roughness
- Awareness ulceration and/or blistering.

## Challenges in diagnosis

Differential diagnoses of suspected OLP and the predominant clinical appearances for each condition are outlined in Box 4.

Box 4 Differential diagnosis of suspected OLP

- OLLs
- Oral lupus erythematosus
- Oral graft versus host disease
- Frictional keratosis
- Leukoplakia including proliferative verrucous leukoplakia
- Mucous membrane pemphigoid
- Linear IgA disease
- Erythema multiforme
- Pemphigus vulgaris
- Paraneoplastic autoimmune multiorgan syndrome.

### Biopsy

It has been suggested that a diagnosis of OLP can be made in asymptomatic patients with typical reticular and bilateral buccal striations without the need for a biopsy.^28^^,^^37^ For all other presentations, incisional biopsies are undertaken in secondary care.

The differential diagnosis of oral lichenoid inflammation includes OLP, oral lichenoid reactions, oral lupus erythematosus and chronic graft versus host disease (Box 4). The appearance of the oral lesions, clinical history (including extra-oral symptoms/signs) and results of special investigations are considered together to reach a diagnosis. The role of histopathology in discerning OLP from OLLs can be very limited due to the mostly indistinguishable features. However, subtle histological differences that may be identified include deep perivascular lymphocytic infiltrate in oral lupus, and even in lichenoid reactions.^4^^,^^38^ The value of histopathological review by an oral pathologist should not be overlooked and is recommended by World Health Organisation (WHO) as the gold standard.^16^

Histopathology is used to distinguish OLP from other immune-mediated mucosal diseases (Box 4). When the clinical presentation is compatible with blistering disorders and/or desquamative gingivitis, the diagnosis must be confirmed with both standard haematoxylin and eosin staining and direct immunofluorescence given the implications on management and prognosis for these conditions. Notably, patients with mucous membrane pemphigoid are referred for ophthalmology review irrespective of history of ocular symptoms due to the potential for subclinical conjunctival involvement and potentially sight-threatening scarring (Box 5).

Box 5 Diagnosis

- It has been suggested that OLP can be diagnosed on clinical grounds alone in patients with typical bilateral buccal white striations
- GDPs should be aware of the diagnostic complexities in cases of atypical, unilateral or asymmetrical lesions, desquamative gingivitis, mucosal blistering, extra-oral involvement and/or constitutional symptoms
- Patients with these features should always be promptly referred to secondary care for consideration of special investigations and appropriate management.

### Patch testing

Lichenoid reactions to dental materials are most commonly seen with amalgam,^39^ although there are reported cases of lichenoid reactions to acrylics, resins and composites.^40^ The role of patch testing for suspected OLLs to dental materials has seen conflicting results in the literature. Concerns have been raised about the validity of the test itself in view of differential reactivity between skin and oral mucosa.^41^ Patch testing should be considered only in conjunction with the clinical presentation. For example, it may prove as a helpful adjunct in patients with an extensively amalgam-restored dentition and widespread lichenoid lesions before embarking on complex and costly dental treatment. On the other hand, patch testing is rarely necessary for lesions in direct contact with an amalgam restoration.^39^ Access to patch testing in primary care is limited.

## Challenges in management

**Fig. 5:**
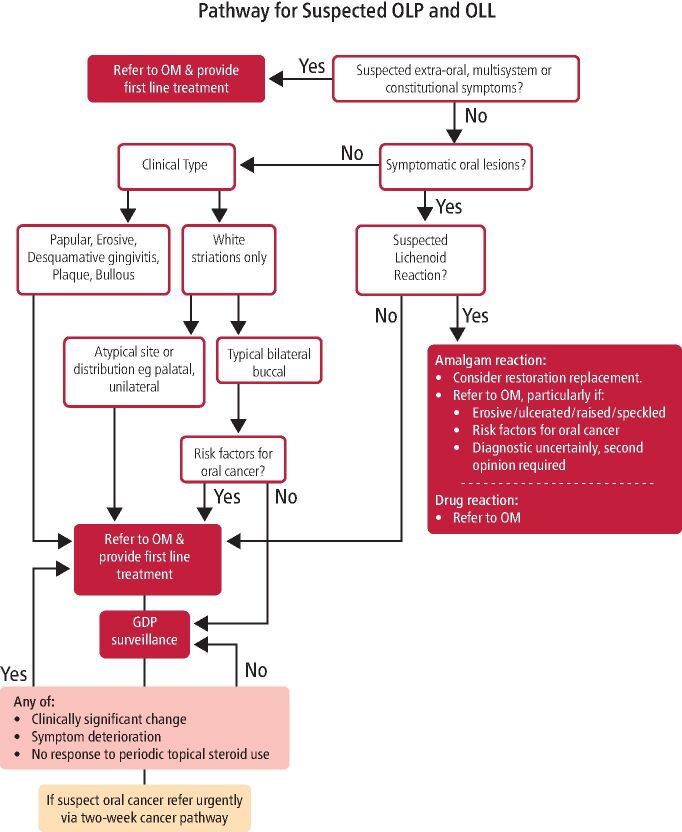
Management of OLP in primary dental care

It is recognised that GDPs will have varying degrees of clinical experience with OLP/OLLs and this should be considered when evaluating the appropriateness of a referral to secondary care. The key points of this paper and a more detailed decision-making tool (Fig. 5) are intended to guide GDPs in the referral and management of suspected OLP/OLLs. If any doubt exists, a referral to secondary care should be made.

### Malignant transformation

A small proportion of OLP and OLLs demonstrate malignant transformation to oral squamous cell carcinoma (0.5-2.5% over five years).^21^ The malignant potential of OLP and OLLs has been long debated, but a recent consensus report by the WHO Collaborating Centre for Oral Cancer has listed both OLP and OLLs as potentially malignant disorders.^16^ OLLs may have a higher transformation rate than idiopathic OLP.^42^ A systematic review by Giuliani *et al.* found the overall malignant transformation rate of OLP and OLLs to be approximately 1.37% and 2.43%, respectively.^1^ Another review found the malignant transformation rate of OLP to be 1.14% and marginally higher for OLLs, but the authors concluded these values were likely underestimations of the true potential of malignant transformation.^42^ Risk factors for malignant transformation were tongue lesions, smoking, alcohol consumption, atrophic-erosive lesions, hepatitis C infection^42^ and female sex.^1^ All patients with OLP/OLLs should be monitored by a dental practitioner. Changes to the typical pattern of disease should instigate a referral to secondary care for consideration of biopsy to assess for dysplasia or malignancy. In cases of suspected malignancy, patients must be referred urgently through the two-week cancer pathway.

### Oral lichen planus

Management of OLP is aimed at symptom control, thus minimising impact on daily activities and improving quality of life. Dentists in primary care can initiate first-line treatment for patients with suspected OLP (see ‘first line treatments' section). Assessment of the impact on quality of life is often integral to the risk-benefit analysis of any pharmacological treatment. The impact on quality of life may be measured with validated tools such as the COMDQ.^43^

### Oral lichenoid lesions

OLLs may present atypically when compared to idiopathic OLP and histological diagnosis in secondary care is often required. In the case of a suspected lichenoid reaction to dental materials, the proximity/direct contact of the lesion(s) with the restoration is often sufficient to support a diagnosis without the need for patch testing. Amalgam replacement results in improvement for over 90% of cases of probable lichenoid reactions to amalgam.^44^ Clinical association between the mucosal lesion and amalgam, together with a positive patch test, was found to be a good predictor of lesion regression.^44^ On the other hand, some evidence supports the replacement of amalgam with an alternative dental material regardless of patch test findings.^45^^,^^46^^,^^47^ Further, restoration replacement may be justified by the higher malignant transformation potential in OLLs compared to OLP, reported by a systematic review.^48^ A risk-benefit analysis of the risk of malignant transformation versus tooth prognosis post-restorative treatment is required. Lesion resolution is unlikely to occur before three months following restoration replacement.^45^

Suspected lichenoid drug reactions should be referred to secondary care for diagnostic confirmation and liaison with the prescribing physician regarding drug substitution where appropriate. Drugs from a similar class can be expected to cause a reaction.^27^

### Monitoring

Close surveillance of OLP and OLLs is critical because of the potential for malignant transformation. GDPs are very well-placed to detect early oral cancer. It has been shown that opportunistic screening of high-risk individuals by GDPs may be a cost-effective screening tool for oral cancer.^6^ There is no universally accepted monitoring interval, although two to four recall appointments per year is deemed a reasonable frequency.^37^ We suggest that the monitoring interval should be a minimum of six months in primary care. The appearance of mucosal disease should be documented at every appointment, if possible, with supporting clinical photographs.

Monitoring in secondary care is dependent on disease severity. Validated tools, such as the Oral Disease Severity Score, may be used to monitor clinically significant change.^49^ Primary and secondary care practitioners have a joint role in monitoring and identifying clinical changes that warrant biopsy to exclude dysplasia or malignancy (Box 6).

Box 6 Long-term care and risk of malignant transformation

- GDPs play a key role in the monitoring of OLP/OLLs
- Secondary care may discharge a patient with OLP/OLLs to their GDP for continued care and monitoring with the expectation of a re-referral in the event of clinically significant changes
- GDPs should monitor OLP/OLL patients at routine appointments even if under specialist care. Patients should be counselled on oral cancer risk and modifiable risk factors, including smoking cessation and alcohol consumption within recommended limits.

### First line treatments

Asymptomatic OLP/OLLs do not require treatment (other than consideration to removal of suspected trigger in OLLs). Mild symptoms may be controlled by avoidance of substances that exacerbate symptoms, for example, strongly flavoured foods and sodium lauryl sulphate-containing oral hygiene products. Oral hygiene and control of periodontal disease is critical, particularly in patients presenting with desquamative gingivitis.

Where simple conservative measures and topical analgesics are insufficient for symptom control, therapeutic management can be commenced in the form of topical steroids. Topical analgesics and anti-inflammatory treatments suitable for prescribing in primary care are outlined in the Scottish Dental Clinical Effectiveness (SDCEP) guidelines (Table 2 and Table 3, respectively) and the British National Formulary Dental Formulary.

**Table 2:** Topical analgesics available in primary care for symptomatic relief (adults), based on SDCEP Drug Prescribing for Dentistry guidelines^50^

Preparation	Send	Label
Benzydamine mouthwash, 0.15%	300 ml	Rinse or gargle using 15 ml up to every 1½ hours as required
Benzydamine oromucosal spray, 0.15%	30 ml	Four sprays onto affected area up to every 1½ hours
Lidocaine ointment, 5%	15 g	Rub sparingly and gently on affected area. Do not use for 30 minutes before eating or drinking
Lidocaine spray, 10%	50 ml	Apply sparingly as required with a cotton bud. Do not use for 30 minutes before eating or drinking

**Table 3:** Preparations available for prescribing off label in primary care, based on SDCEP Drug Prescribing for Dentistry Dental Clinical guidelines^50^

Preparation	Send	Label
Hydrocortisone oromucosal tablets	2.5 mg	One tablet dissolved next to lesion four times daily until healed
Betamethasone soluble dispersible tablets	500 micrograms	Dissolve one tablet in 15 mls of water to make a mouthwash. Rinse around mouth for 5 minutes before spitting out, do not swallow. Use up to four times daily. Do not eat, drink or brush teeth for 30 minutes after use
Clenil Modulite (beclometasone pressurised inhalation)	50 micrograms/metered inhalation	1-2 puffs sprayed onto ulceration twice daily

In primary care, hydrocortisone oromucosal tablets, betamethasone mouthwash and beclomethasone inhaler used as an oral spray can be prescribed off-label (Table 3) and should be accompanied by written patient instructions. The SDCEP's *Drug prescribing for dentistry* guidance includes advice on off-label prescribing. An exemplar patient leaflet for betamethasone mouthwash can be found on the British and Irish Society for Oral Medicine website, together with a general patient information leaflet on OLP.^51^

Antimicrobial mouthwashes, such as chlorhexidine mouthwash, can be helpful, as oral hygiene adjuncts in cases of highly symptomatic disease interfering with optimal toothbrushing.

### Management options in secondary care

In secondary care, alternative topical steroids may be prescribed off-label, including potent/very potent steroid ointments, such as clobetasol or fluocinolone.^52^ Topical steroids ointments may be applied to the gingivae via custom-made trays. A systematic review found topical betamethasone valerate, clobetasol-17-propionate and fluocinonide to be effective in the treatment of OLP when compared with placebo.^53^ On the other hand, a Cochrane review did not support superiority of any topical steroid preparation over another.^54^ There is evidence to support the use of topical calcineurin inhibitors (for example, tacrolimus)^55^ but with only very low-certainty evidence, suggesting that topical tacrolimus may be superior at resolving pain compared to topical corticosteroids.^54^

Where adequate disease control is not achieved with topical therapies, systemic therapies are often commenced in secondary care. This includes periodic courses of systemic prednisolone and prophylactic treatments with disease-modifying antirheumatic drugs, such as hydroxychloroquine, azathioprine and mycophenolate mofetil. Patients on anti-proliferative immunosuppressive therapies (for example, azathioprine and mycophenolate mofetil) will be largely managed in secondary care. However, GDPs should be aware of the increased risk of infection and malignancy associated with these medications.

## Challenges in referral

GDPs are in the unique position of screening oral mucosal health in the general population. OLP/OLLs are likely to make up only a small proportion of a GDP's patient base. Small patient numbers, diverse presentations, differential diagnoses, risk of malignant change and restricted prescribing options for symptomatic patients makes referral to OM departments an important part of patient care.

Referrals must be via the appropriate local pathway. To reduce delays, GDPs should keep login details updated for any electronic systems. The referral should include a provisional diagnosis of suspected OLP or OLLs supported by relevant findings of a detailed history and examination, such as the suspected trigger of OLLs if appropriate.

Challenges can also arise post-referral as OLP cases are typically not prioritised as urgent. GDPs have a responsibility to manage symptomatic patients in the interim. In the event of symptom interference with oral intake, an explicit request to expedite the referral should be made.

Additionally, OLP patients with stable and low-grade disease are typically discharged back to their GDPs for long-term surveillance and care, with the understanding that re-referral should be made in the event of concerning clinical changes or poor symptom control. This can only be achieved if baseline findings are well-documented/photographed, allowing longitudinal comparisons.

## Conclusion

The GDP is ideally placed to screen for and be the first point of contact for patients with OLP and OLLs. Diagnosis and management of these conditions are often complicated by challenges and pitfalls. This paper has highlighted key principles of appropriate management in primary care and appropriate referral to OM departments.
